# Verbalizations Affect Visuomotor Control in Hitting Objects to Distant Targets

**DOI:** 10.3389/fpsyg.2017.00661

**Published:** 2017-04-27

**Authors:** Raimey Olthuis, John Van Der Kamp, Simone Caljouw

**Affiliations:** ^1^Center for Human Movement Sciences, University Medical Centre Groningen, University of GroningenGroningen, Netherlands; ^2^MOVE Research Institute Amsterdam, Faculty of Behavioural and Movement Sciences, Vrije Universiteit AmsterdamAmsterdam, Netherlands

**Keywords:** semantics, motor control, goal-directed movement, perception and action, illusion, language, cognitive motor control

## Abstract

There is a long-standing proposal for the existence of two neuroanatomically and functionally separate visual systems; one supported by the dorsal pathway to control action and the second supported by the ventral pathway to handle explicit perceptual judgments. The dorsal pathway requires fast access to egocentric information, while the ventral pathway primarily requires allocentric information. Despite the evidence for functionally distinct systems, researchers have posited important interactions. This paper examines evidence to what degree the interaction becomes more important when target-identity, the perception of which is supported by the ventral stream, is verbalized during the execution of a target-directed far-aiming movement. In the experiment reported here participants hit balls toward distant targets while concurrently making explicit perceptual judgments of target properties. The endpoint of a shaft served as the target, with conditions including illusory arrow fins at the endpoint. Participants verbalized the location of the target by comparing it to a reference line and calling out “closer” or “further” while propelling the ball to the target. The impact velocity at ball contact was compared for hits toward three shafts of lengths, 94, 100, and 106 cm, with and without verbalizations and delays. It was observed that the meaning of the expressed words modulated movement execution when the verbalizations were consistent with the action characteristics. This effect of semantic content was evident regardless of target visibility during movement execution, demonstrating it was not restricted to movements that rely on visual memory. In addition to a direct effect of semantic content we anticipated an indirect effect of verbalization to result in action shifting toward the use of context-dependent allocentric information. This would result in an illusion bias on the impact velocity when the target is embedded in a Müller-Lyer configuration. We observed an ubiquitous effect of illusory context on movement execution, and not only when verbalizations were made. We suggest that the current experimental design with a far-aiming task where most conditions required reporting or retaining spatial characteristics of targets for action over time may have elicited a strong reliance on allocentric information to guide action.

## Introduction

The evidence that actions and explicit perceptual judgments can depend on different visual information has been available for years (Goodale and Milner, 1992, 1995; Milner and Goodale, 2008). It is presumed that the dorsal pathway, that emanates from the primary visual cortex (V1) and projects dorsally to the posterior parietal cortex, is involved when performing actions in interaction with objects, at least when vision is fully available during movement execution. Online control of movements typically requires fast access to egocentric information, specifying absolute spatial characteristics of the target object relative to the actor. Grasping a cup and bringing it to the mouth, for example, requires the continuous pick up of instantaneous information about the location of the cup in relation to the drinker’s body (i.e., egocentric information). By contrast, the ventral pathway, which projects ventrally from V1 to the inferotemporal cortex, is more involved in perceptual judgments such as object recognition and identification. It primarily requires allocentric information, specifying spatial object information relative to the surrounding objects. For example, information of a container’s handle in relation to its base (i.e., allocentric contextual information) allows its identification as a coffee cup. To allow for recognition of objects over time, information for perceptual judgments needs to remain available for longer durations than information for action, which is only relevant in real-time and not available to conscious awareness (Goodale and Milner, 1995; Milner and Goodale, 2008).

Evidence for this parallel organization of the use of visual information comes from neuropsychological demonstrations showing that brain damage can have dissociated effects on explicit perceptual judgments and action (for a review see Goodale and Westwood, 2004). Moreover, behavioral studies of healthy subjects using geometrical illusions also support the proposal for functionally distinct visual systems for perceptual judgments and action (van Doorn et al., 2007; Bruno et al., 2008; Ganel et al., 2008; Stöttinger et al., 2010, but see for a recent debate Kopiske et al., 2016 and Whitwell and Goodale, 2016). The observation that illusions, most notably the Müller-Lyer illusion, can deceive perceptual judgments while visually controlled movements (often pointing) toward the same object are less affected, is interpreted as evidence for a distinction between the two visual systems. This influence of the contextual elements of geometrical illusions (i.e., the fins of the Müller-Lyer illusion) implies the use of context-dependent allocentric information for explicit perceptual judgments, whereas immunity to the illusion implies the use of context-independent egocentric information.

In the last decade, research has focused less on the dissociation and more on the interplay between the two visual systems (e.g., Schenk and McIntosh, 2009). For example, the ventral system appears to support action more when the action cannot be guided by instantaneous available visual information (i.e., online). In other words, actions become more subserved by the ventral system when vision is occluded before movement initiation and actions must rely on visual memory. Accordingly, the relative immunity of actions to illusions disappears, suggesting that action, when based on visual memory, becomes dependent on allocentric information (Westwood et al., 2000). Similarly, it has also been suggested that dependency on allocentric information, and thus an increased contribution of the ventral system, occurs when action is brought under explicit cognitive control (Rossetti and Régnier, 1995; Willingham, 1998; Keele et al., 2003). Cognitive motor control refers to situations where cognitive strategies (e.g., verbalization, goal setting, mental imagery) are used to guide movement performance and skill acquisition. Cognitive control may be involved in the control of actions by retrieving semantic knowledge. Verbally expressing words related to the task, or presenting words that hold semantic content for the performer, can directly impact the ensuing movement. For example, Gentilucci and Gangitano (1998) found that the kinematics of reaching and grasping movements toward rods, which carried labels with the adjectives ‘long’ or ‘short,’ were altered in line with the semantic content or meaning of the label. Yet, also more subtle semantic cues are seen to influence actions. For instance, when grasping a neutral object with the word ‘apple’ printed on it, larger hand apertures were found than when grasping an object of the same physical size with the word ‘grape’ printed on it (Gentilucci et al., 2000; Glover and Dixon, 2002). These changes in movement kinematics correspond to the meaning of the presented words and thus indicate that semantic content can have a direct influence on movement execution, even when the actions are made under full vision.

Apart from its direct influence on action, semantics may also have a more indirect impact through a change in the information that is exploited for action. Although several authors suggested that allocentric information comes to play a more prominent role when movements are controlled more consciously (Willingham, 1998; Keele et al., 2003; Glover, 2004), empirical evidence is scarce. We found only one study showing that semantics may invoke the use of allocentric information (Rossetti and Régnier, 1995; see also Rossetti, 1998). In fact, this shift in information-use was suggested to be similar to changes that occur when adding a delay. The authors examined pointing movements toward objects with and without verbalization and with and without a delay between presentation of the object and the onset of the movement. They argued that verbalizing meaningful properties of the target object changes movement kinematics based on an increased reliance upon context-dependent allocentric information. Participants pointed to one of six targets presented along a horizontal arc. Concurrently, the participants were required to either make a meaningless utterance (i.e., without reference to the spatial configuration) or verbalize a number (i.e., 1–6) that was associated with the target location. They found that with an unrelated utterance movement errors were aligned in the direction of the movement (i.e., irrespective of the neighboring targets), suggesting movement control based on egocentric information. By contrast, in the verbalization condition, movement errors were aligned in the direction of the neighboring targets, suggesting control based on allocentric information. In fact, the later errors were similar to the delay condition without verbalization. Although this supports the idea that movements with verbalizations may lead to the exploitation of allocentric information, it does not prove it. The research indicates the type of movement error, but does not delineate the type of information that underlies these errors. Manipulation of allocentric information by presenting a target for aiming surrounded by contextual elements of a geometrical illusion can help to shed further light on this issue.

The present study therefore investigates whether verbalizations of task properties directly affects the movement kinematics in the direction of the semantic content of the verbalization and incites the use of context-dependent or allocentric information. To this end, participants propelled balls toward the end of a shaft, which was presented in isolation or embedded within the Müller-Lyer illusion. The task was performed with and without verbalization and with and without a delay between presentation and movement onset. In the verbalization condition, participants judged the endpoint of the shaft relative to a reference line by calling “closer” or “further” while performing the action task. In the delay conditions the target configuration was removed before a go signal indicated movement initiation. We expected (1) a direct effect of the verbalization with differences in the movement kinematics that mirrored the semantic contents “closer” versus “further”; (2) an indirect effect of the verbalization with enhanced differences in the movement kinematics that result from using context-dependent or allocentric information, that is, an enhanced illusory bias; and (3) that these direct and indirect effects of verbalization dissipated when visual information about the target object was available at all times (i.e., no-delay condition).

## Materials and Methods

### Participants

Eight right-handed participants (2 female) volunteered in this experiment (mean age 24.8 +/- 3.0 years). They all reported normal or corrected to normal vision. The local ethics committee approved the study and all participants gave their written informed consent prior to participation.

### Apparatus and Procedure

**FIGURE 1:**
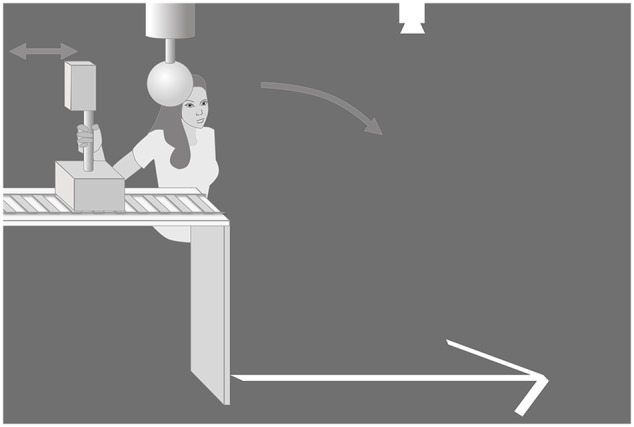
**Experimental set-up**.

**Figure 1** shows the experimental set-up. General features of the hitting task are also reported in previous publications (Caljouw et al., 2006, 2011). Participants were seated on a chair with the handle of the hitting device in their right hand. The hitting device comprised a vertically oriented rod (32 cm), mounted on one end to a trolley that could freely slide, with little frictional resistance, back and forth along a straight trackway and on the other end to a Perspex block (2.5 cm wide) at the same vertical position as the ball. Meanwhile, the mid portion of the vertical rod was the handle of the hitting device (**Figure 1**). The ball (diameter 7.5 cm) was suspended from above in mid-air at eye level by a rod with an air circuit that reduces air pressure and, therefore, held the ball in place by suction. To strike the ball participants moved the hitting device horizontally along the track. The start position of the hitting device was close to the ball, so the hitting device was first pulled away and then pushed toward the ball in a smooth bi-phasic sliding movement. When the block struck the ball it detached from the air circuit and was propelled across the target lines that were projected with a beamer on the ground. This set-up allowed the participant to control the velocity of the hitting device at ball contact, and correspondingly the ball projection distance, by adjusting the amplitude of the backswing (Caljouw et al., 2006).

Participants visited the lab five times within a week. They were allowed to perform two sessions in 1 day with at least 2 h rest in between. To improve their hitting skills, participants started with three practice sessions of 50 min each. During each practice session approximately 550 balls were hit to targets at different distances that were randomly presented. The target was indicated by the endpoint of a line that was projected on the ground. The practice target lines were within a range that could be reached with the hitting device, from 75 to 125 cm, with steps of 1.09 cm (i.e., 46 different landing locations). Participants were instructed to aim the ball as accurately as possible toward the end of the line. During the practice trials, a successful hit was granted when the ball landed on a coin (1.5 cm) that was positioned at the endpoint of the line. If so, the experimenter gave a compliment and the number of successful hits was presented on a digital screen. To ensure motivation throughout the practice sessions, a reward was given to the participant with the most successful hits in a session. In the last 5 min of each practice session participants practiced hitting (˜50) balls with occluded vision from approximately 3 s before and during movement execution. They were instructed to look at the line, close their eyes, count to three, and then hit the ball with their eyes closed to the remembered target location.

In the experiment, factorial combinations of verbalization (no verbalization, verbalization) and visual condition (no delay, 3 s delay) resulted in four experimental blocks. In each experimental session two blocks, consisting of 100 hitting trials each, were performed. The presentation order of these four blocks was counterbalanced between participants by means of a Balanced Latin Square design. Before the experimental session started participants practiced for 5 min (e.g., they projected about 50 balls to the practice target lines, see above), and between the two experimental blocks within a session participants rested for 5 min and performed 10 practice trials. Practice trials were performed with knowledge of results to motivate participants to remain accurate during the experimental trials.

In each condition participants projected the ball to the endpoint of the shaft of one of the five target configurations (e.g., short shaft of 94 cm, medium shaft of 100 cm, long shaft of 106 cm, medium shaft with tails-in, and medium shaft with tails-out). The tails of the illusion were 36 cm long and made an angle of 45° with the shaft. All lines (shaft and tails) were 2 cm wide.

In the no-delay blocks, participants were instructed to initiate their hitting movement the moment the target configuration appeared. The configuration was presented for 2 s, which was enough time to provide full vision of the target during movement initiation and execution. Often the configuration disappeared just before ball-target contact, so knowledge of results was not always available during the experimental trials.

In the delay blocks there was a 3 s delay between target presentation and movement initiation. A target configuration was presented for 2 s and then disappeared, 3 s later a beep signal indicated for the participant to start their hitting movement. Thus, immediately before and during movement initiation the target configuration was not visible and actions had to rely on visual memory of the target configuration.

In the verbalization blocks participants were required to call ‘dichterbij’ (closer) or ‘verderweg’ (further) while executing the hitting movement, depending on the perceived location of the target (e.g., the endpoint of the shaft of the target configuration) relative to the endpoint of the reference line. The reference line was a tailless line of 100 cm (i.e., the medium shaft) and presented before the appearance of the target configuration. At the start of each verbalization trial the reference line was presented for 1 s and then replaced by one of the five target configurations. When the endpoint of the shaft of the presented target configuration was perceived to be further than the endpoint of the earlier presented reference line, participants were instructed to report ‘verderweg’ (further) and when the endpoint of the shaft of the presented target configuration was estimated to be closer than the endpoint of the earlier presented reference line, participants were instructed to report ‘dichterbij’ (closer), while at the same time initiating the hitting movement to project the ball to the target configuration. Thus, in the delay-verbalization block participants reported their estimation after the beep signal indicating movement initiation. When the endpoint of the shaft was estimated at the same location as the endpoint of the reference line participants were asked to make a balanced choice between the two verbal responses ‘dichterbij’ (closer) and ‘verderweg’ (further).

### Data Collection and Analysis

To analyze the perceptual estimate of the required landing location, the verbal response (closer or further) in each verbalization trial was noted during the experiment. To analyze the outcome of the hitting movement, two infrared emitting diodes (IRED’s) were positioned: one on the hitting device (to determine the velocity at impact) and the second above the ball on the rod (to determine the moment of impact). A 3D registration system (Optotrak) registered the position of the IREDs at a sampling frequency of 500 Hz. The moment of ball contact (i.e., defined as the first inflection point in the velocity profile) and impact velocity (i.e., defined as the velocity of the hitting device at the moment of ball contact) were computed from the 3D positions of the two IREDs with a second order recursive Butterworth low pass filter with a cut-off frequency of 10 Hz. Caljouw et al. (2010) previously showed that in this task impact velocity is a good and reliable estimate of the ball landing location. The differences in verbal reports between the configuration conditions were analyzed with Wilcoxon signed-rank tests. To assess the effect of verbalizations and delay on impact velocity a RM-ANOVA was performed with Target (short versus long), Delay (delay versus no-delay), and Verbalization (with versus without) as within-subjects factors. To assess whether verbalization and delay incites the use of allocentric information a RM-ANOVA was performed with Target (fins in versus fins out), Delay (delay versus no-delay), and Verbalization (with versus without) as within-subjects factors. Where the sphericity assumption of RM-ANOVA was violated Huynh-Feldt correction was applied.

## Results

### Direct Effects of Verbalization

We selected the responses to the short shaft and long shaft without tail configurations to assess the effect of verbalization on impact velocity (and thus landing location). We hypothesized that verbalizing “closer” and “further” would affect the impact velocity in the direction conveyed by the semantic content of the pronounced words, especially when a delay was introduced.

#### Verbal Responses

**FIGURE 2:**
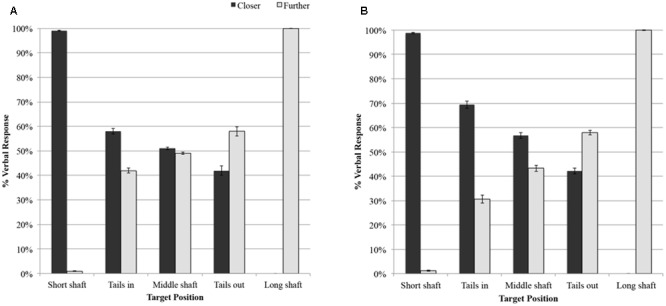
**The percentage of “closer” and “further” responses for each configuration (A)** in the no-delay condition and **(B)** in the delay condition.

**Figure 2** illustrates the percentage of “closer” and “further” responses for each target configuration in the delay and no-delay conditions, respectively. When aiming for the long shaft configuration all participants verbalized “further” in all trials. For the short shaft configuration 7 out of 8 participants verbalized “closer” in all trials. One participant verbalized “further” when aiming for the short shaft configuration in two trials (out of 20) in the delay condition and in one trial (out of 20) in the no-delay condition. The Wilcoxon signed-rank test revealed a significant difference (*z* = -2.636, *p* < 0.01) in the number of times participants called closer for the short shaft (Mdn. = 40, Q1 = 38.5, Q3 = 40) compared with the long shaft (Mdn. = 0, Q1 = 0, Q3 = 0) condition.

#### Impact Velocity

**FIGURE 3:**
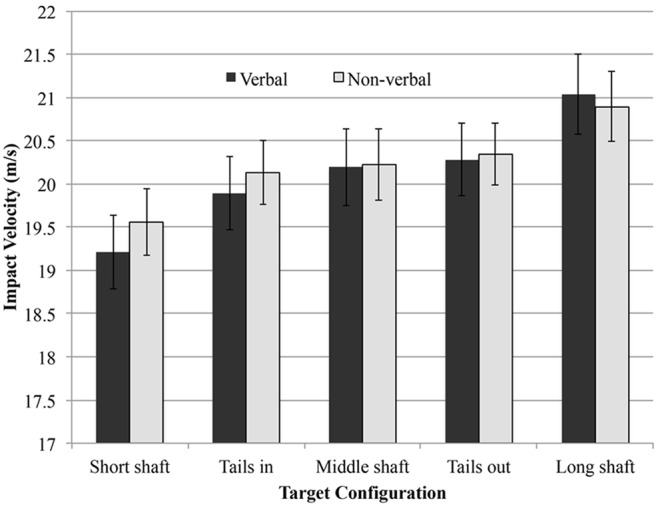
**The mean impact velocity and standard error bars for the five target configurations for both verbalization conditions**.

**FIGURE 4:**
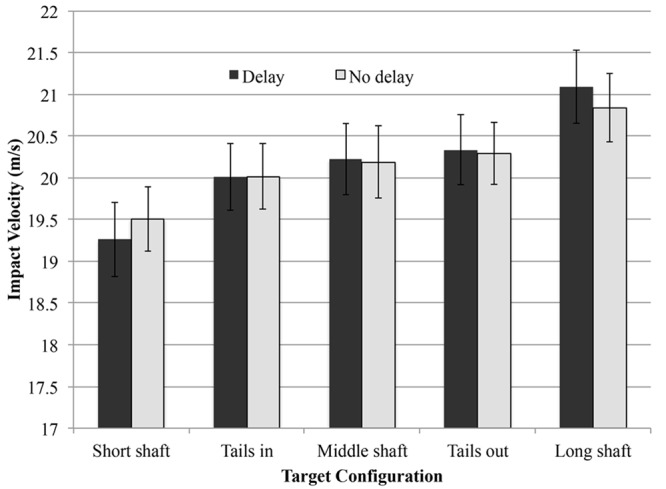
**The mean impact velocity and standard error bars for the five target configurations for both delay conditions**.

A 2 (Delay: delay, no-delay) by 2 (Verbalization; verbal, non-verbal) by 2 (Target: long, short) ANOVA with repeated measures on all factors revealed a significant main effect of Target [*F*(1,7) = 122.18, *p* < 0.001, $\eta_{p}^{2}$ = 0.95] and interaction effects of Verbalization × Target [*F*(1,7) = 10.93, *p* < 0.05, $\eta_{p}^{2}$ = 0.61] (see **Figure 3**) and Delay × Target [*F*(1,7) = 7.39, *p* < 0.05, $\eta_{p}^{2}$ = 0.51] (see **Figure 4**). There was no significant three-way interaction effect. As expected, balls were hit with a larger impact velocity to the long shaft configuration than to the short shaft configuration. This effect was more pronounced in the verbal condition compared with the non-verbal condition and in the delay condition compared with the no-delay condition.

#### Further Analyses

Responses to the medium shaft without tail configuration were selected to assess whether merely articulating the words “closer” or “further” affected the impact velocity. The length of the medium shaft configuration was the same as the length of the reference line; hence, participants were most likely to make a random choice between the “further” and “closer” verbal responses. When reporting the distance for the medium shaft configuration without tails the responses “closer” and “further” were relatively equally distributed at 53.8 and 46.2%, respectively (see **Figure 2**). The Wilcoxon signed-rank test revealed no significant difference in the number of times (out of 40) participants called “closer” (Mdn. = 20, Q1 = 19.25, Q3 = 21.50) compared with “further” (Mdn. = 19.50, Q1 = 18.00, Q3 = 20.75) for the medium shaft. To compare the effect of verbalizing the word “closer” or “further” on the impact velocity when hitting to the medium shaft without tails configuration we performed a 2 (Response: “closer,” “further”) by 2 (Delay: delay, no-delay) ANOVA with repeated measures on both factors. Neither significant main effects, nor significant interaction effects were revealed.

### Indirect or Illusion Effects of Verbalization

Responses to the targets with the tails-in and tails-out configurations were selected to assess whether verbalization with regard to target location would increase the illusion bias on impact velocity.

#### Verbal Responses

Participants verbalized “closer” more frequently in the tails-in condition (Mdn. = 23.5, Q1 = 18.5, Q3 = 33.25) compared to the tails-out condition (Mdn. = 18.5, Q1 = 8.75, Q3 = 21.50). The Wilcoxon signed-rank test revealed that this effect was significant (*z* = -2.252, *p* < 0.05).

#### Impact Velocity

A 2 (Delay: delay, no-delay) by 2 (Verbalization; verbal, non-verbal) by 2 (Target: tail-in, tails-out) ANOVA with repeated measures on all factors revealed a significant main effect of Target [*F*(1,7) = 43.69, *p* < 0.001, $\eta_{p}^{2}$ = 0.86], but no other significant main or interaction effects. Balls were hit with a higher impact velocity to the tails-out than the tails-in configuration.

## Discussion

In this study participants performed a visually guided action; they propelled a ball to a visual target defined by the end of a shaft projected on the ground. Concurrently, participants made an explicit perceptual judgment; they verbalized the location of the target by comparing it to a reference line and calling out “closer” or “further” while they propelled the ball to the target. To examine the direct effect of the semantic content (or meaning) of this verbalization, the impact velocity at ball contact was compared for hits toward shafts of different lengths with and without verbalization. In addition, we anticipated that next to a direct effect of semantics, an indirect effect of verbalization might occur with the control of the action shifting toward the use of context-dependent allocentric information. This would result in an illusion bias on the impact velocity when the target is embedded in a Müller-Lyer configuration. A delay between target presentation and movement initiation was introduced to study whether the semantic effects dissipate when the target remains visible during movement execution.

### Direct Effects of Verbalization

The current study provides further support for the contention that task-relevant semantic information directly affects action kinematics. That is, the semantic content or meaning of the explicitly articulated judgments about the target location (“closer” and “further”) systematically modulated the impact velocity, and thus the force by which the ball is projected to the target. This modulation of impact velocity was aligned with the location expressed in the verbalization: “closer” calls for short target shafts and “further” calls for long shafts resulted in lower and higher velocities, respectively, compared to conditions in which no verbalizations were required. This finding is congruent with previous studies (Gentilucci et al., 2000; Glover and Dixon, 2002), but adds to this literature in showing that target-related semantic information is not only effective for reaching and grasping movements for targets in peripersonal space but also for actions directed toward targets in extrapersonal space. Previous work reported that target-related words systematically modulated the maximum hand aperture (Gentilucci et al., 2000) and the end point of a reaching movement (Kritikos et al., 2012). This resulted in the contention that specifically the *positioning* of the fingers and hands in online controlled (closed-loop) actions cannot withstand direct influences of semantics (Kritikos et al., 2012). Here, we extend those observations to the *speed* or *force* of ballistic (open-loop) far-aiming movements.

The verbalization of “closer” and “further,” however, did not significantly affect impact velocity when aiming for the medium shafts. We had expected that merely articulating the words would affect the hitting action in a similar manner to the short and long shafts, even though the meaning of the articulated words may be irrelevant to the task in this condition (see also Bartolo et al., 2007). However, in the current study, the direct verbalization effects were only significant when the semantic content was aligned (or congruent) with the task at hand; that is, when the length of the shaft was longer or shorter than the reference line and the participants correspondingly called “further” and “closer.” Yet, for the medium shaft length, the verbalization effect was not found. In this condition, the target shaft and the reference line were of the same length. Perhaps by forcing them to call out “further” or “closer” even when they perceived the lines to be of the same length, the verbalization was less meaningful for the participants, as they did not sincerely believe in the word they uttered. This would be in line with the work of Fargier et al. (2012), who showed that unrelated action verbalization had no direct influence on movement kinematics. The small number of participants is a limitation of this work. A small sample size may adversely affect the statistical power to detect relatively small effects in this study. As a consequence, small but possibly consistent differences in direction when calling “further” or “closer” in the medium shaft condition may have gone unnoticed. This indicates that we cannot rule out an effect of verbalization in this condition with a larger sample size. In future work, judgments and verbalizations should be more clearly dissociated to further investigate the conditions under which direct effects of verbalization do and do not occur. For example, continued research may further assess whether the effect of words is amplified when the difference between the reference line and the target line is more obvious and certainty about the estimation increases.

The direct effects of verbalization were found when the target was removed from view prior to movement initiation thereby preventing the instantaneous use of visual information about the target location during movement execution. It was expected that these effects would diminish when visual information about the target was salient and available all the time. After all, previous studies on grasping objects bearing printed words demonstrated that semantic information (via words) affected the kinematics only in the early portion of the movement, that is, before online corrections modify grip aperture to the real target width (Gentilucci et al., 2000; Glover and Dixon, 2002). Contrary to our expectations, we observed an effect of verbalization on the impact velocity of the movement, not only when a delay was introduced between target presentation and movement initiation, but also under conditions of full vision of the target during movement execution. This might indicate that fast online visual control to annul the inaccuracies is prevented in this far-aiming task (see more detailed discussion below). However, this seems unlikely given our previous observations that participants are able to make rapid adjustments of the hitting movement in response to unexpected far target location perturbations after movement initiation (Caljouw et al., 2006, 2011). Possibly the fast dorsal movement control acts in parallel with cognitive control process that underlie the direct effects of verbalization (see Willingham, 1998).

Even though the participants were not required to explicitly judge and verbalize target-relevant properties in the no verbalization condition, impact velocity was stronger affected by target distance in the delay or memory guided condition compared to the no-delay condition. It is not particularly clear why this effect occurred. The explicit labeling of targets in previous conditions may have resulted in self-instruction, especially in the delay condition without online vision of the target, where inner speech might have led participants to increase and decrease impact velocity when hitting to the far and near targets, respectively. To prevent this verbal overshadowing in future studies an additional distracting task can be used.

### Indirect or Illusion Effects of Verbalization

Besides the direct effects of verbalization in line with its semantic content, we also expected that verbalization would affect the action kinematics indirectly through increased reliance on context-dependent allocentric information. This should result in an illusion bias when the target is presented within a Müller-Lyer tail configuration

Indeed, we found that impact velocity was affected by the illusory context. Contrary to expectations, the illusion bias was ubiquitous and observed independent of temporal delay and verbalization. This contrasts with other studies, which typically reveal a stronger bias in memory guided actions than in visually guided movements (for a review see Bruno et al., 2008). The more systematic illusion bias in the present study suggests that the current task and/or design constraints facilitated the use of context-dependent allocentric information. First, the far-aiming characteristics may hamper the instantaneous use of visual information during movement execution. In line with the present findings, an illusion bias on movement outcome under full vision conditions is typically observed in far-aiming tasks, such as throwing balls (van der Kamp and Masters, 2008; Shim et al., 2014) or sliding disks (van der Kamp et al., 2009). These findings seems best explained by participants using allocentric information because they (implicitly) define the target location relative to the visual surroundings, especially when the target itself is not always visually specified (e.g., when aiming for the empty space in the corner of a goal in handball). Although the task constraints of far aiming may set up reliance on context-dependent allocentric information, its use is by no means compelled. In a previous study, in which participants more extensively practiced the hitting task, the existence of an illusion bias under full vision conditions could not be substantiated (Caljouw et al., 2010).

A second explanation for the ubiquitous use of allocentric information in this study may have been the cognitive involvement that was required in most conditions: in 75% of the trials, participants either made verbalization or guided the hitting based on memory (i.e., delay conditions). This may have invoked an increased involvement of the ventral system, including a penetration in movement control under conditions (i.e., no verbalization and no delay) that normally do not engage the ventral system. In other words, the ubiquitous illusion bias may have arisen because participants were relatively conscious of movement execution. Also van Doorn et al. (2007) reported that participants were more inclined to exploit allocentric information to guide actions, if the actions were performed in the context of other more deliberate conditions.

## Conclusion

We found that verbalizing action characteristics (i.e., target location) directly influences movement execution in a far-aiming task, even when veridical visual information about the location is available during movement execution. However, these effects predominantly emerged when verbalizations are aligned with the task characteristics. We also show that the use of context-dependent allocentric information is not confined to guidance of memory-based actions. However, to what degree verbalization (indirectly) enhances reliance on allocentric information remains unclear. The current study does certainly not rule out such a scenario, but can also not definitely prove it. Future research must shed further light on the particular task constraints that are most influential in this respect.

## Ethics Statement

This study was carried out in accordance with the Code of Ethics laid down by the Faculty of Human Movement Sciences of the Vrije Universiteit with written informed consent from all subjects. All subjects gave written informed consent in accordance with the Declaration of Helsinki. The protocol was approved by the local ethical committee human movement sciences.

## Author Contributions

SC and JVDK made substantial contributions to conception and design, acquisition of data, and analysis and interpretation of data. RO made substantial contribution to analysis and interpretation of data. SC, JVDK, and RO participated in drafting the article and revising it critically for important intellectual content; and give final approval of the version to be submitted and are accountable for all aspects of the work in ensuring that questions related to the accuracy or integrity of any part of the work are appropriately investigated and resolved.

## Conflict of Interest Statement

The authors declare that the research was conducted in the absence of any commercial or financial relationships that could be construed as a potential conflict of interest.
